# Construction of Polymer Electrolyte Based on Soybean Protein Isolate and Hydroxyethyl Cellulose for a Flexible Solid-State Supercapacitor

**DOI:** 10.3390/polym11111895

**Published:** 2019-11-17

**Authors:** Zhiyu Xun, Shoupeng Ni, Zhenhua Gao, Yanhua Zhang, Jiyou Gu, Pengfei Huo

**Affiliations:** 1 Materials Science and Engineering College, Northeast Forestry University, Harbin 150040, China; 15963729607@163.com (Z.X.); nisp2019@163.com (S.N.); gaozh1976@163.com (Z.G.); zhangyanhua@nefu.edu.cn (Y.Z.); 2 Key Laboratory of Bio-based Materials Science & Technology (Northeast Forestry University), Ministry of Education, Harbin 150040, China

**Keywords:** gel polymer electrolyte, soybean protein isolate, hydroxyethyl cellulose, solid-state supercapacitors

## Abstract

Supercapacitors are a very active research topic. However, liquid electrolytes present several drawbacks on security and packaging. Herein, a gel polymer electrolyte was prepared based on crosslinked renewable and environmentally friendly soybean protein isolate (SPI) and hydroxyethyl cellulose (HEC) with 1.0 mol L^−1^ Li_2_SO_4_. Highly hydrophilic SPI and HEC guaranteed a high ionic conductivity of 8.40 × 10^−3^ S cm^−1^. The fabricated solid-state supercapacitor with prepared gel polymer electrolyte exhibited a good electrochemical performance, that is, a high single electrode gravimetric capacitance of 91.79 F g^−1^ and an energy density of 7.17 W h kg^−1^ at a current density of 5.0 A g^−1^. The fabricated supercapacitor exhibited a flexible performance under bending condition superior to liquid supercapacitor and similar electrochemical performance at various bending angles. In addition, it was proved by an almost 100% cycling retention and a coulombic efficiency over 5000 charge–discharge cycles. For comparison, supercapacitors assembled with commercial aqueous PP/PE separator, pure SPI membrane, and crosslinked SPI membrane were also characterized. The obtained gel polymer electrolyte based on crosslinked SPI and HEC may be useful for the design of advanced polymer electrolytes for energy devices.

## 1. Introduction

Supercapacitor (SC) is considered as an electrochemical energy storage device with a great potential due to its high-power, long cycle life, low environmental impact, and high safety at present [1]. Recently, the development of flexible solid-state supercapacitors fabricated with polymer electrolytes that can be used in wearable electronic devices has attracted more attention.

It is well known that the energy density of a supercapacitor is determined by the specific capacitance and squared operating voltage window [2,3]. To improve the energy density, tremendous efforts have been devoted to developing novel electrode materials to elevate the specific capacitance [4,5,6,7,8,9,10]. Apart from the electrodes, electrolyte is one of the key components of a supercapacitor, that can significantly affect the operating voltage as well as lifespan and safety of supercapacitors [11,12,13]. The supercapacitors assembled with liquid electrolytes have the disadvantages of electrolyte leakage, high packaging costs, and usually being toxic or corrosive, which limits their application for portable electronic devices [14].

The design and development of polymer electrolytes have been attracting much attention due to excellent safety, stability, flexibility, etc., which make them widely used in various electronic devices such as lithium-sulfur batteries, lithium-metal batteries, lithium-ion batteries, fuel cells, and supercapacitors [15,16,17,18,19,20,21,22]. Piana et al. reported poly(ethylene oxide) (PEO)/Li_1.5_A_l0.5_Ge_1.5_(PO_4_)_3_ hybrid solid polymer electrolytes for super Li^+^ ion conducting ceramic with high ionic conductivity and excellent Coulombic efficiency (>99.5%) under high current regimes [16]. A 3D GPE based on poly(vinylidene fluoride-hexafluoro propylene) (PVDF-HFP) with polymerized pentaerythritol tetrakis-divinyl adipate was prepared and could suppress Li dendrites growth in lithium-sulfur batteries [18]; Wang et al. prepared epoxy based adhesive polymer electrolyte with high ionic conductivity (>10^−2^ S cm^−1^) for a solid-state supercapacitor [20]. In summary, polymer electrolytes can replace liquid electrolytes and serve as both separator and electrolyte with a high ionic conductivity at ambient temperature, good mechanical strength, excellent stability, and a wide potential window in energy devices [23]. Polymer electrolytes can be classified into solid polymer electrolytes (SPEs) and gel polymer electrolytes (GPEs). SPEs mostly deliver low ionic conductivities (10^−8^–10^−5^ S cm^−1^) and poor interfaces with electrodes, resulting in deteriorated cycle performance [24,25,26,27]. GPEs have been extensively investigated because they can deliver an ionic conductivity as high as liquid electrolyte and provide a variety of potential advantages, including good interfacial contact with electrodes, higher safety and adaptability, and flexibility for flexible energy storage devices [28,29,30].

GPE can be prepared by assembly of polymer skeleton and supporting liquid electrolyte. The polymer substrate plays an important role in a GPE. However, non-degradable synthetic polymer materials in GPE face several disadvantages and are not environmentally friendly. Hence, it is imperative to develop biopolymer electrolytes by using natural polymers, which have gained more and more attention, owing to their abundance in nature, low cost, friendliness to the environment, and potential as substitute for some petrochemicals [31]. Biomass materials—such as cellulose, chitosan, soybean protein, etc.—are of concern for their regenerability and degradation in nature.

As a biomass material, soybean protein isolate (SPI), a byproduct of the soybean oil industry, has been applied in many fields including energy storage due to its renewability, biocompatibility, biodegradability, processability, and film-forming capacity [32], and the ionic protein containing different types of amino acids can be used to transport ions for energy storage devices [33]. Zhu et al. prepared porous membranes with SPI and PVA via electrospinning as separators for lithium ion batteries [34]. A composite GPE was constructed from porous polydopamine spheres and SPI membrane to suppress Li dendrite growth and trap manganese ions for a high-performance Li battery [35]. These studies demonstrate that SPI can be used as a polymer matrix for polymer electrolyte; furthermore, SPI contains abundant polar functional groups to obtain excellent hydrophilicity, which provides the membrane with a good electrolyte affinity. However, the application remains substantially limited due to the high-water sensitivity, undesirable processability, and inferior mechanical strength [36]. A lot of investigations on modification of SPI have been carried out to solve the problems mentioned above [37,38,39]. In our previous work, a series of crosslinked membranes was prepared by SPI crosslinked with ethylene glycol diglycidyl ether (EGDE) as the crosslinker and it was found that the crosslinked structure could improve the water resistance of SPI/EGDE membrane, but it also reduced the ionic conductivity of the pure SPI film and electrochemical performance of the supercapacitor [40].

To improve the decline in electrochemical performance due to the crosslinked membrane and maintain good water resistance, a highly hydrophilic polymer can be introduced into the crosslinked structure to construct a composite system. Hydroxyethyl cellulose (HEC), a derivative of cellulose, has a wide range of sources [41]. HEC has excellent hydrophilicity and been widely used as a water-soluble additive. Simultaneously, it has other characteristics, such as film-forming capacity similar to cellulose. Moreover, HEC can interact with polar groups such as amino groups and carboxyl groups on SPI to form a uniform and stable film material [42].

In this study, a series of membranes was prepared by SPI as the polymer matrix, blended with HEC and crosslinked by ethylene glycol diglycidyl ether (EGDE) which is an epoxy compound with low toxicity. The crosslinked membranes were then saturated with 1.0 M Li_2_SO_4_ electrolyte with a large operating potential window to form gel polymer electrolytes (GPEs) and applied in solid-state supercapacitors (SSCs). The comprehensive properties of the membranes, GPEs, and SSCs were systematically investigated. The membrane based on the crosslinked SPI and HEC not only maintains its water resistance, but also improves the ionic conductivity of GPE and electrochemical performance of supercapacitor.

## 2. Materials and Methods

### 2.1. Materials

SPI (96.5 wt %) was purchased from Harbin Gaoke Food Technology Co., Ltd. (Harbin, China). HEC was obtained from Aladdin Industrial Corporation. Glycerin (analytical grade) was acquired from Guangfu technology development Co., Ltd. (Tianjin, China). Activated carbon (AC) and acetylene black (battery grade) were provided by Japan KURARAY (Shanghai, China) and Crisco Chemical Technology Co., Ltd. (Shanghai, China), respectively. EGDE and polytetrafluoroethylene (PTFE) (60 wt %) concentrate were purchased from Aladdin Reagent (Shanghai, China) Co., Ltd. (Shanghai, China). Nickel foam was purchased from Kejing Zhida Co., Ltd. (Shenzhen, China). Anhydrous lithium sulfate and commercial hydrophilic PP/PE composite membrane were received from Saen Chemical Technology Co., Ltd. (Shanghai, China) and Lizhiyuan Battery Co., Ltd. (Taiyuan, China) respectively.

### 2.2. Preparation of Gel Polymer Electrolyte Based on Crosslinked SPI and HEC

SPI powder was dispersed in deionized water and stirred constantly to form a suspension. Glycerol was added and the pH of suspension was adjusted to 10, after which the suspension was stirred for 30 min at room temperature followed by being heated at 85 °C under constant stirring for 30 min. The suspension was then cooled down to room temperature and ultrasonicated for 10 min. Finally, a 5 wt % SPI solution was obtained. The HEC powder was dissolved in deionized water to form a 5 wt % solution simultaneously. The SPI solution and HEC solution were mixed for 2 h. The crosslinker EGDE was dropped into the solution and stirred for 1 h. In order to maintain a similar degree of crosslinking of each membrane, equal amount of crosslinker EGDE was added in the membranes of the same quantity. The homogeneous solution was cast onto a dry flat glass plate and dried at 50 °C for 24 h. The membrane based on crosslinked SPI and HEC was peeled off and recorded as MCM-H. SPI membrane named MCM-S and crosslinked SPI membrane named MCM-E were prepared in a similar process described above. For comparison, the commercial hydrophilic PP/PE membrane was used as a separator and named MCM-C. The component information was listed in Table 1. All the membrane samples were immersed in 1.0 M Li_2_SO_4_ electrolyte to form GPEs named GPE-H, GPE-S, GPE-E, and GPE-C, respectively.

### 2.3. Fabrication of SSCs

**Figure 1:**
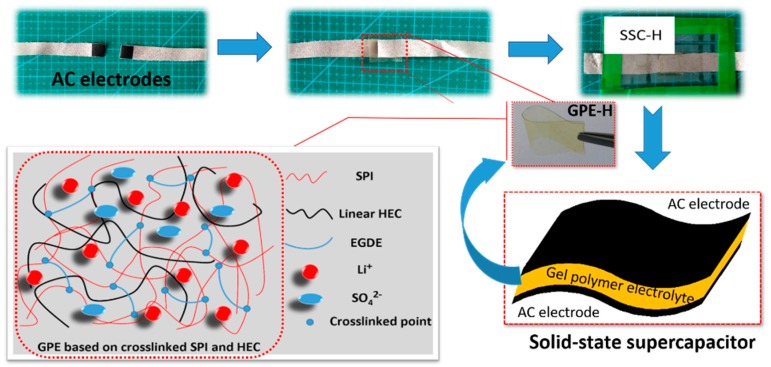
Schematic illustration of the assembly process of SSC and schematic diagram of crosslinked based on SPI and HEC.

Firstly, the AC electrodes were prepared by 80 wt % AC powder, 10 wt % acetylene black and 10 wt % PTFE, and each electrode was 4.1 mg. The SSCs were then assembled as the following sandwiched structure: AC electrode//GPE//AC electrode and simply packaged. The process was described as shown in Figure 1. For comparison, a SSC with GPE-C was also fabricated by the same method and recorded as SSC-C. The flexibility of supercapacitor was named SSC-H’ and tested by assembling polymer electrolyte and electrodes with area of 3 cm^2^ (1 × 3 cm).

### 2.4. Characterization of Membrane Samples

The ATR-FTIR spectra of all the samples were recorded from 500 to 4000 cm^−1^ using a Spectrum One FTIR spectrophotometer (Nicolet Co., Madison, WI, USA). XRD of samples were evaluated with a D/max-2 200 diffractometer (Rigaku International Corporation, Tokyo, Japan). X-ray radiation was generated from a Cu-Kα source at an accelerating voltage of 40 kV and a current of 30 mA. The diffraction data were collected from 5° to 50° with a step interval of 0.02°. Thermogravimetric analysis (TGA) of samples were analyzed using a Netzsch 209 F3 TGA instrument (NET Scientific Instruments Trading (Shanghai) Co., Ltd., Shanghai, China) under a Nitrogen atmosphere from 30 °C to 600 °C at a heating rate of 10 °C min^−1^.

### 2.5. Electrolyte Uptake of Membranes

Membrane samples (1.5 × 4.0 cm) were dried at 80 °C for 24 h to a constant weight which was recorded as *W_dry_*. The dry membrane was immersed in 1.0 M Li_2_SO_4_ for 30 min at room temperature, followed by wiping with filter paper for removal of excess Li_2_SO_4_ solution on the surface, the weight of the wet membrane was recorded as *W_wet_*. The electrolyte uptake (EU) was calculated by the equation.

$$EU\left(Electrolyte\;uptake\right)\left(wt\;\%\right)=\frac{W_{wet}-W_{dry}}{W_{dry}}\times 100\%$$

Five replicates were performed for each membrane.

### 2.6. Ionic Conductivity of GPEs

The ionic conductivity (*σ*, S cm^−1^) of the GPEs can be calculated by the equation.

$$\sigma =\frac{L}{R_{b}\times S}$$

where *L* (cm) is the thickness of the GPE, *S* (cm^2^) is the effective interface area between GPE and stainless-steel electrode. *R_b_* (ohm) is the bulk resistance of the GPE and measured by electrochemical impedance spectroscopy (EIS) measurement with a potential amplitude of 10 mV and a frequency range from 1 Hz to 100 kHz by using electrochemical workstation (CHI660B, Shanghai, China). In order to establish the relationship between the ionic conductivity of GPE and the electrochemical performance of the corresponding SSC, only one set of data for each GPE and corresponding solid-state supercapacitor is used and discussed.

### 2.7. Electrochemical Characterization of SSCs

The electrochemical performance of the SSCs with different GPEs was investigated in a two-electrode system by EIS, cyclic voltammetry (CV) and galvanostatic charge–discharge (GCD) techniques by using electrochemical workstations (CHI660B, Shanghai, China). The EIS spectrum was used to analyze the impedance behavior of the single cell with frequency range from 0.01 Hz to 100 kHz at the amplitude of open-circuit voltage. CV tests of the solid-state supercapacitors were conducted within a stable potential window (0–1.5 V) at different scan rate (10, 20, 50, 100, 200 mV s^−1^). GCD characterization was conducted within a stable potential window (0–1.5 V) at different current density (1, 2, 5, 10 A g^−1^). GCD measurement was used to determine the capacitance and the cycle performance of cells. The single electrode gravimetric specific capacitance (*C_sp_*, F g^−1^) was calculated from the GCD curves according to the equation.

$$C_{sp}=\frac{4I}{m}\times \frac{dt}{dv}$$

where *I* is the constant current (A), *m* is the AC mass of two electrodes (g), *dv*/*dt* (V s^−1^) is the slope of the fitting straight line to the discharge curve excluding the IR drop. The energy density (*E_cell_*, W h kg^−1^) and power density (*P_cell_*, W kg^−1^) of SSC were calculated as.

$$E_{cell}=\frac{CV^{2}}{2}=\frac{C_{sp}V^{2}}{8}$$

$$P_{cell}=\frac{E_{cell}}{\Delta t}$$

where *V* is the cell voltage without voltage drop, Δ*t* is the discharge time.

The electrochemical cycling life of the SSC-H was investigated for 5000 charge and discharge cycles at a current density of 2 A g^−1^ by using LAND test equipment (LAND, CT3001A, Wuhan, China).

## 3. Results and Discussion

### 3.1. Structural Analysis of Membrane Samples

**Figure 2:**
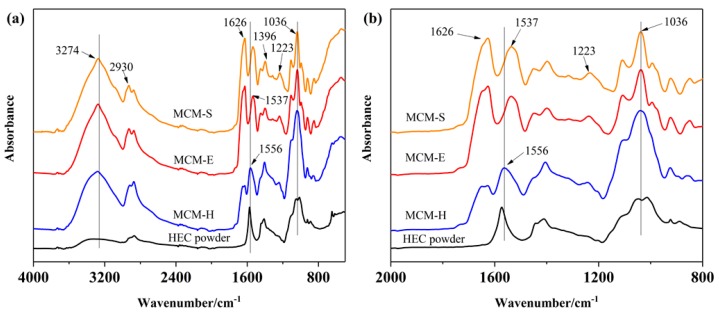
(**a**) ATR-FTIR spectra of HEC powder, MCM-S, MCM-E, MCM-H from 500 to 4000 cm^−1^, (**b**) ATR-FTIR spectra from 800 to 2000 cm^−1^.

The ATR-FTIR spectra of HEC powder, MCM-S, MCM-E, and MCM-H are shown in Figure 2. The adsorption band at 3000–3500 cm^−1^ and about 2900 cm^−1^ were attributed to the free bound –OH and –CH_3_ and –CH_2_– in the prepared membranes, respectively. The absorption peaks of characteristic group of protein were observed at 1626 cm^−1^, 1537 cm^−1^, and 1223 cm^−1^ and correspond to amide I (C=O stretching vibration), amide II (N–H bending vibration), and amide III (C–N stretching and N–H bending vibration), which were consistent with a previous report [43]. The COO– and –C–NH_2_ absorption peaks could be observed at 1396 cm^−1^ and 1036 cm^−1^, respectively [44,45]. From Figure 2b, it can be observed that the introduction of HEC, the absorption peak of amide II (1537 cm^−1^) shifted to 1556 cm^−1^, which was due to the hydrogen bond interaction between HEC and SPI.

**Figure 3:**
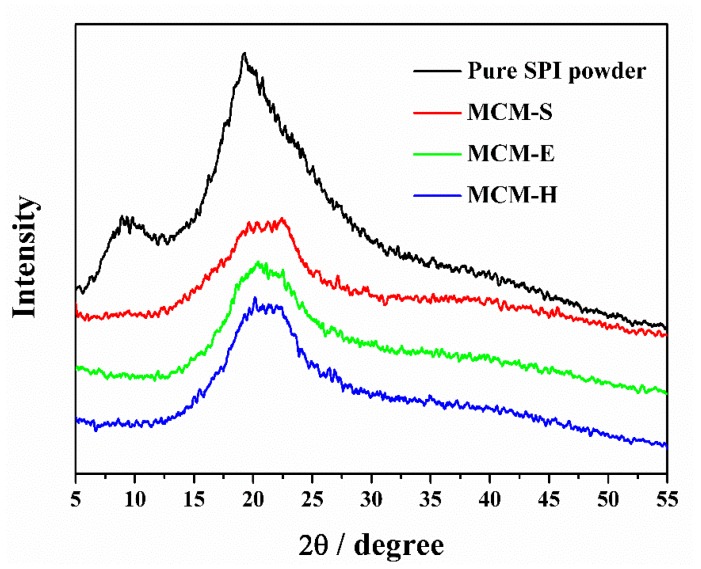
XRD patterns of SPI power and membrane samples.

XRD patterns of pure SPI power and membrane samples are shown in Figure 3. Characteristic diffraction peaks at 2θ ≈ 9.3° and 19.3° of the pure SPI power represent the α-helix and β-sheet structures of SPI secondary structure, respectively [46]. It is obvious that these two characteristic diffraction peaks can be found in MCM-S. With the addition of EGDE, the characteristic absorption peak at 2θ ≈ 9.3° of MCM-E disappeared, which indicates that a chemical reaction occurred between EGDE and SPI. The XRD curves of MCM-E and MCM-H are almost the same, indicating that high compatibility between SPI and HEC.

### 3.2. Thermal Stability

**Figure 4:**
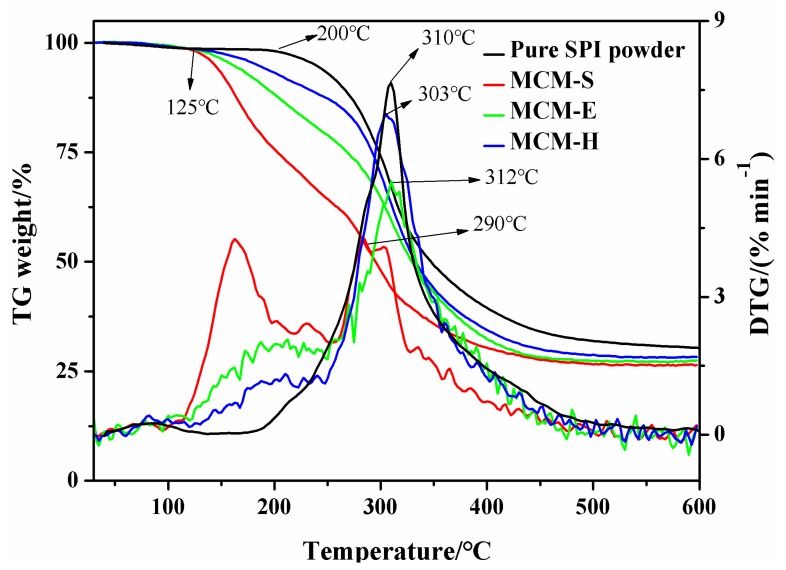
TG and DTG patterns of SPI powder and membrane samples.

Thermal stability of samples was investigated using TGA. The TGA curves recorded from 30 °C to 600 °C are shown in Figure 4. Only one decomposition stage can be observed for thermal decomposition of the SPI powder. The initial degradation and the maximum decomposition rate of SPI powder occurred at around 200 °C and 310 °C. Initial decomposition of modified SPI membranes occurred at around 125 °C, which is lower than that of the SPI powder, which may be due to partial degradation of the SPI molecular structure caused by KOH and glycerol. Decomposition temperature of MCM-H is higher than MCM-S and MCM-E from the beginning to the end of thermal decomposition. Maximum decomposition rate temperature of MCM-E is significantly higher than MCM-S and is even higher than that of SPI powder. Although the maximum decomposition rate temperature of MCM-H is slightly lower than that of MCM-E, it is still higher than that of MCM-S, which indicates that the crosslinked structure can significantly improve the thermal stability of MCM-H membrane.

### 3.3. Electrolyte Uptake, Water Resistance of Membrane Samples, and Ionic Conductivities of GPEs

**Figure 5:**
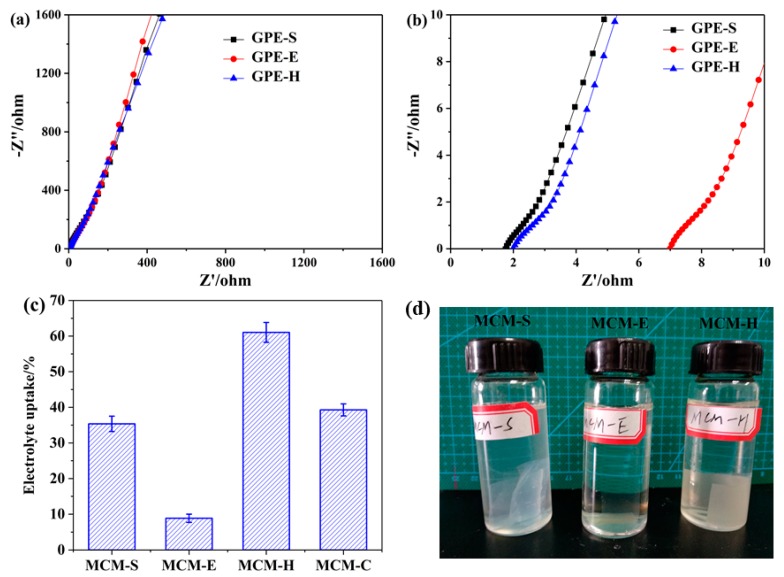
(**a**) Nyquist plots of GPEs, (**b**) the high frequency region of the Nyquist plots, (**c**) the electrolyte uptake of GPEs, and (**d**) water resistance of membrane samples.

The Nyquist plots of GPEs are shown in Figure 5a,b. The Ionic resistance (*R_b_*) can be determined by the intersection of the Nyquist plot and the Z’ axis in high frequency area. The ionic conductivities of GPEs can be calculated and displayed in Table 2. The lower ionic conductivity of GPE-E resulted from the consumption of the hydrophilic amino groups in the SPI via the crosslinking reaction with EGDE. GPE-H has a higher ionic conductivity and electrolyte uptake than GPE-S and GPE-E. The addition of hydrophilic HEC compensates for the loss of hydrophilic functional groups due to crosslinking reaction and due to HEC facilitating the transport of electrolyte ions, which can be obviously observed from the electrolyte uptake results in Figure 5c. SPI membrane has a good ionic conductivity due to the good hydrophilicity as well as ionic property to facilitate the ion transport of the polymer electrolyte. This is an important factor in the selection of SPI as a polymer matrix of polymer electrolyte. Furthermore, comparison between GPE-H and GPE-C was carried out. GPE-C exhibits a higher ionic conductivity due to the high porosity facilitating the transport of ions.

As a natural polymer material, SPI has poor water resistance. To study the water resistance of SPI membranes, the membrane samples were submerged in deionized water for 24 h as shown in Figure 5d. MCM-S has been excessively swollen and almost lost its mechanical strength. Conversely, MCM-E and MCM-H exhibited better stability in deionized water.

### 3.4. Electrochemical Performance of SSCs

**Figure 6:**
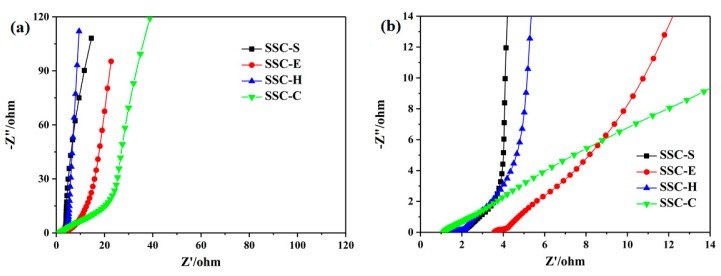
(**a**) Nyquist plots of SSCs in the frequency range of 0.01 Hz–100 kHz, (**b**) the high frequency region of the Nyquist plots.

The electrochemical performance of SSCs was explored by EIS, CV and GCD techniques. For comparison, the electrochemical performance of SSC with GPE-C was also investigated. Nyquist plots of SSC-X (SSCs with different GPEs) in the frequency range of 0.01 Hz-100 kHz was shown in Figure 6. The intercept with real axis (Z’ axis) in the high frequency region (Z” = 0) reflects equivalent series resistance (*R_s_*) of SSC cells, which is associated with the ionic resistance of the electrolyte, the intrinsic resistance of the electrode material and the contact resistance at the interface of electrode material [47,48,49]. An approximate semicircular behavior at the high to medium frequency region refers to the interfacial charge transfer resistance (*R_ct_*), which is related to the process at the electrolyte/electrode interface [50]. A slope line at medium frequency region is related to diffusion of the electrolyte, its length depends on the frequency of electrolyte ions transporting between electrode and polymer electrolyte [51]. The straight line nearly vertical to the axis at low frequency region indicates excellent capacitive behavior of the SSC cells [52]. Electrostatic diffusion capacity and overall capacitance behavior of SSCs assembled by SPI membranes are superior to SSC assembled with GPE-C. Various electrical parameters of SSC-E associated with bulk properties of electrolyte and electrode–electrolyte interface in different frequency ranges are worse than SSC-S. This is because crosslinked structures consume a large number of hydrophilic groups in the SPI and reduce the electrolyte uptake of MCM-E. Conversely, SSC-H exhibits a similar *R_s_* with SSC-S and shows a better capacitive behavior than SSC-S. HEC in GPE-H is beneficial for absorption of liquid electrolyte and the transport of ions, and the moderate swelling of GPE-H caused by HEC improves the interface performance and promotes the transport of ions at the electrode–electrolyte interface. Compared with SSC-H, SSC-C assembled with GPE-C shows a low *R_s_* due to its high ionic conductivity, however, SSC-C exhibits a larger ion transfer resistance between GPE-C and electrodes than SSC-H since MCM cannot be swelled by electrolyte, which results in a worse electrochemical performance than SSC-H.

**Figure 7:**
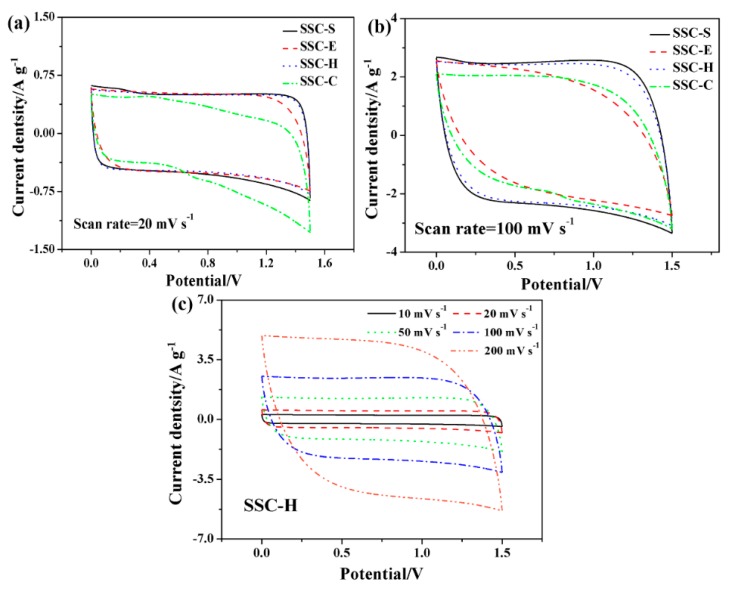
(**a**) CV curves of SSCs at scan rate of 20 mV s^−1^ and (**b**) 100 mV s^−1^, (**c**) CV curves of SSC-H at various scan rates.

CV curves of SSCs at scan rates of 20 and 100 mV s^−1^ are shown in Figure 7a,b, respectively. All curves of SSCs have similar rectangular profiles, symmetric with respect to the X axis and without redox peaks. The CV curves of SSC-C and SSC-E deviate significantly from the rectangle, while those of SSC-S and SSC-H still maintain a good rectangular at high scan rates. In addition, integral area of the curves of SSC-S and SSC-H are almost overlapping, which are far greater than those of SSC-E and SSC-C, demonstrating that the SSC-S and SSC-H exhibit similar capacitance behaviors better than SSC-E and SSC-C. The result further illustrates that the introduction of HEC in GPE-H improved the electrolyte uptake and accelerated the transport of electrolyte ions at the electrode–electrolyte interface, which elevated the capacitance of SSC-H. CV curves of SSC-H at different scan rates (10–200 mV s^−1^), as depicted in Figure 7c, showed only a slight deviation from perfect rectangular even at high scan rate of 200 mV s^−1^, which indicated that the capacitive behavior of SSC-H belongs to electrical double-layer formation and SSC-H had lower internal resistance. HEC in MCM-H accelerates transport of the electrolyte ions and reduces the equivalent series resistance of the GPE-H, which endows SSC with a higher specific capacitance and a better power performance.

**Figure 8:**
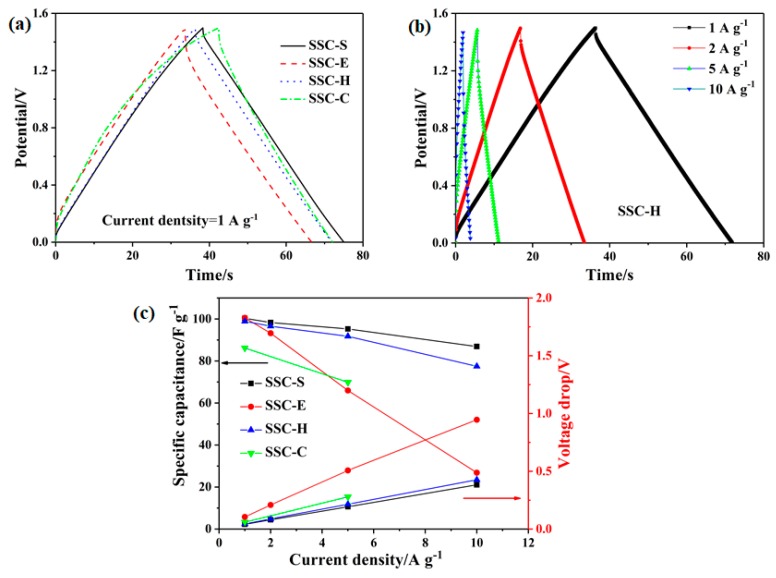
(**a**) GCD curves of SSCs at the current density of 1 A g^−1^, (**b**) GCD curves of SSC-H at various current densities, (**c**) specific capacitance and voltage drop of SSCs at various current density.

GCD curves of SSCs at the current density of 1 A g^−1^ are shown in Figure 8a. The curves of SSC-S and SSC-H exhibited an ideal linear profile, the charge curves were almost perfectly symmetrical with their corresponding discharge curve within the potential window (0–1.5 V). However, the GCD curves of SSC-E and SSC-C deviate from the ideal symmetrical triangle, which indicates that SSC-H has superior electrical double layer capacitance characteristics and reversibility of charge and discharge over SSC-E and SSC-C. The GCD curves of SSC-H at different current densities (1–10 A g^−1^) are shown in Figure 8b. The GCD curve of SSC-H still maintains a symmetrical linear shape even at high current density, which further demonstrates that SSC-H has a good reversibility charge and discharge. *C_sp_* and voltage drop (*V_drop_*, V) of the SSC-X were calculated according to the discharge profile and demonstrated in Figure 8c. The *C_sp_* of SSC-S and SSC-H decreased slightly and the *V_drop_* increased with the increase of current density due to internal resistance of SSCs. However, the *C_sp_* of SSC-E and SSC-C are greatly reduced and the *V_drop_* increase substantially with the increase of current density. This demonstrates that SSC-H has a better reversibility of charge and discharge and small internal resistance, which indicates that GPE-H can accelerates the transport of electrolyte ions and reduces the internal resistance of SSC-H due to the introduction of HEC. The *C_sp_* value of SSC-H was 98.91 F g^−1^ which is much higher than that of SSC-C (86.22 F g^−1^) at a current density of 1 A g^−1^. Even at high current densities of 5 A g^−1^, the *C_sp_* of SSC-H can reach 91.79 F g^−1^, whereas the sharp decay of *C_sp_* of SSC-C with commercial membrane MCM-C is only 69.9 F g^−1^. The *C_sp_*, *E_cell_*, and *P_cell_* of SSC-H were calculated at different current densities and listed in Table 3.

**Figure 9:**
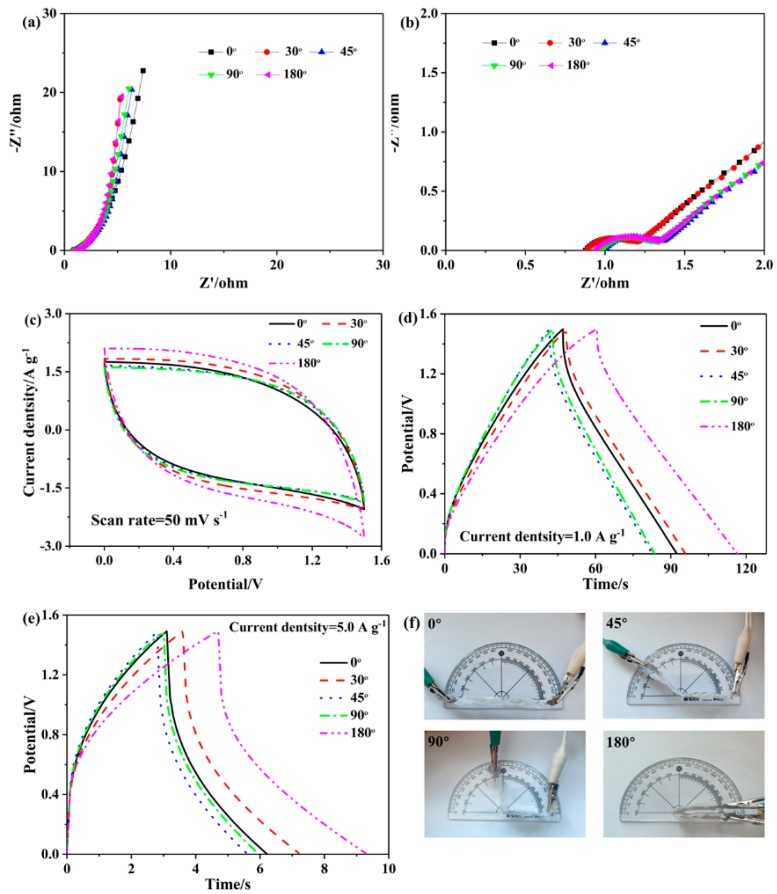
Electrochemical properties of SSC-H under various bending angles. (**a**) Nyquist plots in a frequency range from 0.01 Hz to 100 kHz, (**b**) Nyquist plots in the high frequency region, (**c**) CV curves at a scan rate of 50 mV s^−1^, (**d**) GCD curves at a current density of 1.0 A g^−1^, (**e**) at a current density of 5.0 A g^−1^, and (**f**) picture of various bending angles.

Flexibility is a crucial parameter for SSCs applied in wearable electronic devices and portable devices. The electrochemical performance of SSC-H’ was systematically measured under different bending angles as shown in Figure 9. The resulting CV curves, GCD curves and EIS curves with the bending angles of 0°, 30°, 45°, 90°, and 180° are shown in Figure 9a–e, respectively. Figure 9f is physical pictures of the various angles in the experiment. From the Nyquist plots in Figure 9a,b, it is known that at different angles, there is a small equivalent series resistance in the high frequency region, indicating that the bend has little effect on the equivalent series resistance; a vertical line close to 90° is shown in the low frequency region, indicating that the bend has almost no effect on the interface change between GPE-H and the electrode. The almost overlap CV and GCD curves for SSC-H’ at different bending angles are obtained from the Figure 9c–e. Figure 9b presents the analogous CV responses and area. The as-measured GCD curves in Figure 9d,e show a negligible change at different bending angles at a current density of 1.0 A g^−1^ and 5.0 A g^−1^. The approximate EIS, CV, and GCD curves at different angles reveal good angle stability and ideal flexibility of SSC-H’ for application in flexible electronic devices.

**Figure 10:**
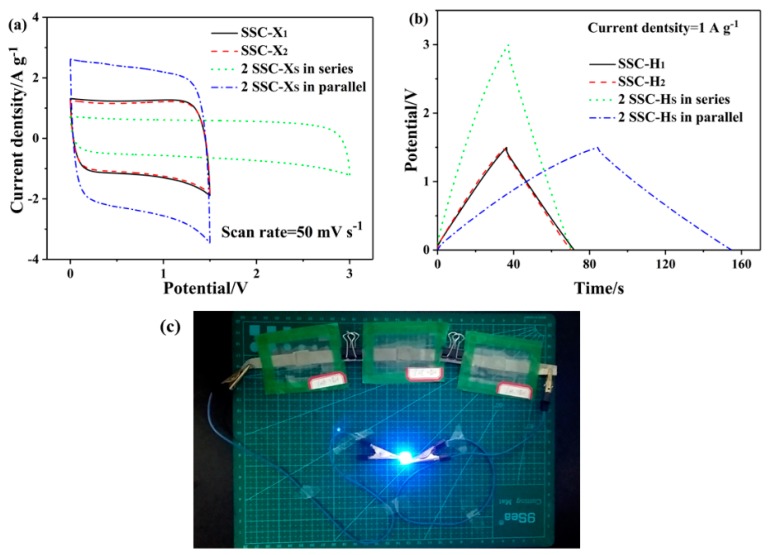
(**a**) CV curves of two SSC-Hs connected in series and in parallel at a scan rate of 50 mV s^−1^, (**b**) GCD curves of two SSC-Hs connected in series and in parallel at current density of 1 A g^−1^, (**c**) photograph of the LED powered by three SSC-Hs connected in series.

Two SSC-H cells connected in series and parallel were evaluated by CV and GCD measurements as shown in Figure 10a,b. Two SSC-H cells could be connected in series or in parallel and obtain a device that outputted approximately twice the potential, or twice the current density and charge–discharge time of the single SSC-H. As shown in Figure 10c, the 2 V light-emitting diode (LED) could be lighted up by three SSC-H cells in series connection, which indicated their good practical prospects.

**Figure 11:**
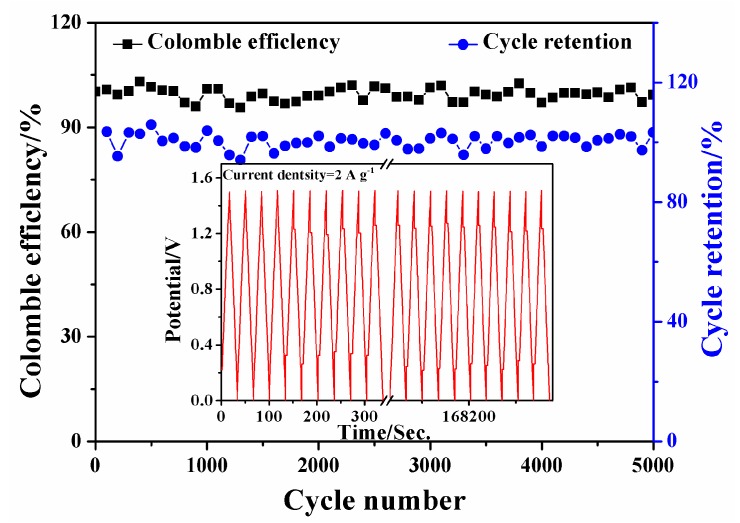
Cycle stability of SSC-H at a current density of 2.0 A g^−1^.

The long cycle life of SSCs is one of the most important factors with regard to its practical application. To investigate the cycling stability, the SSC-H cell was tested at a constant current density of 2 A g^−1^ over 5000 continuous cycles. Figure 11 exhibits the variation of the coulombic efficiency and cycling retention as a function of the charge–discharge cycle numbers and the first 10th and last 10th charge–discharge curves of SSC-H are displayed in the inset of Figure 11. The coulombic efficiency calculated from the ratio of the discharge capacitance to the charge capacitance, maintained almost as high as 100% for 5000 cycles, which indicates that SSC-H has good charge and discharge reversibility. Even though the cycle retention shows a slight fluctuation in the early stage of charge–discharge, it remained almost 100% even after 5000 cycles. The GCD curves still maintain a good symmetrical and linear shape and are almost the same as the initial GCD curves indicating an excellent electric double layer capacitive behavior and cycle stability of SSC-H.

## 4. Conclusions

A transparent, flexible, biodegradable membrane based on crosslinked SPI and hydrophilic HEC was successfully prepared, and the GPE-H was obtained by the membrane saturated with 1 M Li_2_SO_4_. The water resistance of the MCM-E is significantly improved due to the crosslinked structure of the membranes. However, the ionic conductivity of GPE-E is reduced. The addition of the HEC not only improves the water resistance of the MCM-H, but also facilitates the transport of electrolyte ions for GPE-H. The electrochemical performance of SSCs assembled from SPI composite membranes were higher than that of SSC assembled by commercial aqueous PP/PE membrane. SSC-H assembled with GPE-H exhibits a high capacitance of 91.79 F g^−1^ and an energy density of 7.17 W h kg^−1^ at a current density of 5 A g^−1^, which is much higher than SSC-E assembled with GPE-E. According to the experiments at various angles, it was proved that SSC-H’ fabricated with GPE-H has good flexibility. The solid-state supercapacitors also show considerable application performance at series a parallel connection condition. Electrochemical cycle performance of the SSC-H indicated almost 100% cycling retention and near 100% coulombic efficiency over 5000 charge–discharge cycles at 2 A g^−1^. The results obtained in this work confirm that GPEs based on renewable, degradable biomass material are interesting candidates for replacing liquid electrolytes for flexible solid-state devices. Further investigations on regulating composition structure of gel polymer electrolyte based on SPI/HEC system to optimize the mechanical stability and improve the electrochemical performance such as energy density and specific capacitance are currently in progress.

## Figures and Tables

**Table 1:** Component information of the prepared modified membranes.

Samples	SPI (5 wt % Solution, g)	Glycerol (g)	EGDE (g)	HEC (5 wt % Solution, g)
MCM-S ^a^	12	0.3	0	0
MCM-E ^b^	12	0.3	0.12	0
MCM-H ^c^	8.4	0.21	0.12	3.6

^a^ The pure SPI membrane; ^b^ The crosslinked SPI membrane; ^c^ The crosslinked SPI membrane with HEC.

**Table 2:** Electrolyte uptakes of membrane samples and thickness, ionic conductivities of GPEs.

GPE Sample	Thickness of GPEs (cm)	Ionic Conductivity (10^−3^ S cm^−1^)	Membrane Samples	Electrolyte Uptake (%)
GPE-S ^a^	0.0133	7.58	MCM-S	35.35 ± 2.16
GPE-E ^b^	0.0157	2.25	MCM-E	8.84 ± 1.15
GPE-H ^c^	0.0168	8.40	MCM-H	61.03 ± 2.80
GPE-C ^d^	0.0138	9.40	MCM-C	49.27 ± 1.68

^a^ Gel polymer electrolyte with MCM-S; ^b^ Gel polymer electrolyte with MCM-E; ^c^ Gel polymer electrolyte with MCM-H; ^d^ Gel polymer electrolyte with MCM-C.

**Table 3:** Specific capacitance, energy density, and power density of SSCs at different current densities.

Sample	Current Density (A g^−1^)	Specific Capacitance (*C_sp_*, F g^−1^)	Energy Density (*E_cell_*, W h kg^−1^)	Power Density (*P_cell_*, W kg^−1^)
SSC-S ^a^	1	100.35	7.84	771.15
	5	95.27	7.44	5351.76
SSC-E ^b^	1	100.55	7.86	862.68
	5	65.98	5.15	6621.43
SSC-H ^c^	1	98.91	7.73	786.10
	5	91.79	7.17	4780.00
SSC-C ^d^	1	86.22	6.74	786.89
	5	69.9	5.46	5312.43

^a^ Solid-state supercapacitor with GPE-S; ^b^ Solid-state supercapacitor with GPE-E; ^c^ Solid-state supercapacitor with GPE-H; ^d^ Solid-state supercapacitor with GPE-C.
